# Type IV dual left anterior descending coronary artery: a case report

**DOI:** 10.1186/s13104-017-2984-z

**Published:** 2017-12-01

**Authors:** Sahela Nasrin, Fathima Aaysha Cader, M. Maksumul Haq, Masuma Jannat Shafi

**Affiliations:** 1 Department of Cardiology, Ibrahim Cardiac Hospital & Research Institute (ICHRI), 122, Kazi Nazrul Islam Avenue, Shahbagh, Dhaka, 1000 Bangladesh; 2 National Institute of Cardiovascular Diseases (NICVD), Dhaka, Bangladesh

**Keywords:** Coronary vessel anomaly, Dual left anterior descending artery, Bangladesh

## Abstract

**Background:**

Dual left anterior descending (LAD) artery or duplication of LAD is a rarely reported coronary anomaly, consisting of two branches supplying the usual distribution of the LAD. Type IV dual LAD, in which a short LAD arises from the left main coronary artery and a long LAD arises from the right coronary artery is remarkably rare, and has not been reported in a Bangladeshi subject.

**Case presentation:**

We describe the case of a 70-year old Bangladeshi male who presented with breathlessness in the background of a prior inferior myocardial infarction. Coronary angiography revealed an anomalous dual LAD. The short LAD which arose from the left main coronary artery gave off the first septal branch and terminated after giving off a large diagonal branch which continued further down towards the apex. The long LAD arose from the proximal right coronary artery and after traversing a distance, arrived at the interventricular septum, terminating at the apex after giving off diagonal branches. The right coronary artery was totally occluded from its early mid part and well-collateralized with retrograde flow from the left system.

**Conclusion:**

We describe a case with unique variation of dual LAD type IV, which has previously not been described in a Bangladeshi subject thus far. Coronary angiography is vital to determine this coronary anomaly, which is usually detected incidentally on routine angiography for chest pain, sometimes with involvement of significant lesion of other coronary arteries, as in this case.

## Background

Coronary vessel anomalies are a diverse group of congenital disorders with variations in origin, course and termination, and intrinsic coronary arteries [1]. The presence of anomalous coronaries is an important pathological entity that needs to be evaluated in patients presenting with chest pain, as it may lead to myocardial ischaemia, arrhythmia or sudden cardiac death [2, 3]. Nevertheless, not all anomalous coronaries result in signs, symptoms, or complications, and most of them are benign, usually discovered as incidental findings at the time of catheterization [3]. An anatomic variant of anomalous left anterior descending (LAD) artery is termed “dual LAD” or duplication of LAD. It is a rare anomaly, reported in 1% of individuals with otherwise normal hearts [3, 4]. Dual LAD consists of two branches which supply the usual distribution of the LAD [2]. One branch (short LAD) terminates in the proximal aspect of the anterior interventricular sulcus (AIVS). A second, longer branch has a variable course outside the AIVS and returns to the AIVS distally.

We report a case of a 70-year-old Bangladeshi male presenting with breathlessness and a history of prior inferior myocardial infarction (MI), in whom an incidental detection of anomalous LAD of dual origin was found on coronary angiography. Consistent with a type IV dual LAD, this pattern of dual LAD, to the best our knowledge, has not been previously reported in a Bangladeshi subject. He also had occluded right coronary artery (RCA), the culprit lesion, which received retrograde collaterals from the anomalous left system.

## Case presentation

A 70-year old Bangladeshi male presented with exertional shortness of breath and paroxysmal nocturnal dyspnoea for 6 months. He also gave a history of chest pain of sudden onset 6 months prior to this admission, for which he had not sought medical attention. Electrocardiogram showed pathological Q waves in inferior leads and left ventricular hypertrophy. Echocardiography revealed moderate eccentric mitral regurgitation, septal hypertrophy and global hypokinesia with a left ventricular ejection fraction of ~ 35%.

**Fig. 1:**
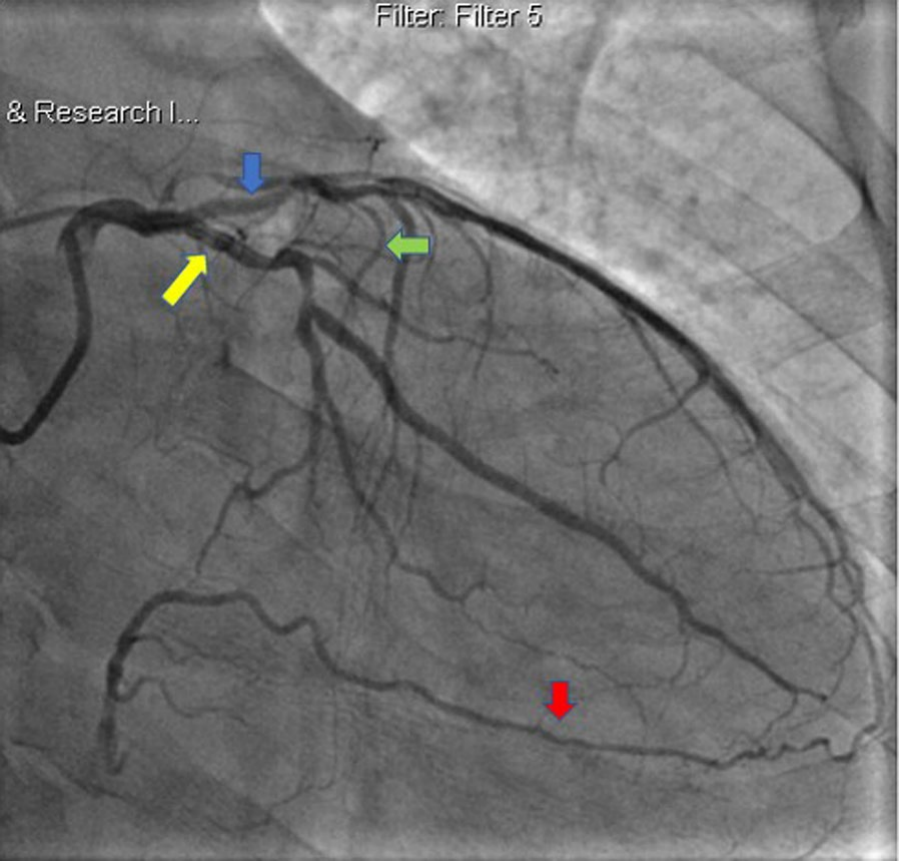
Coronary angiography RAO Caudal view. The left main coronary artery bifurcates into short left anterior descending artery (blue arrow) and left circumflex coronary artery (yellow arrow). The short LAD gives off the first septal branch (green arrow). Retrograde collaterals to the right coronary artery (red arrow) from the left system are also seen

**Fig. 2:**
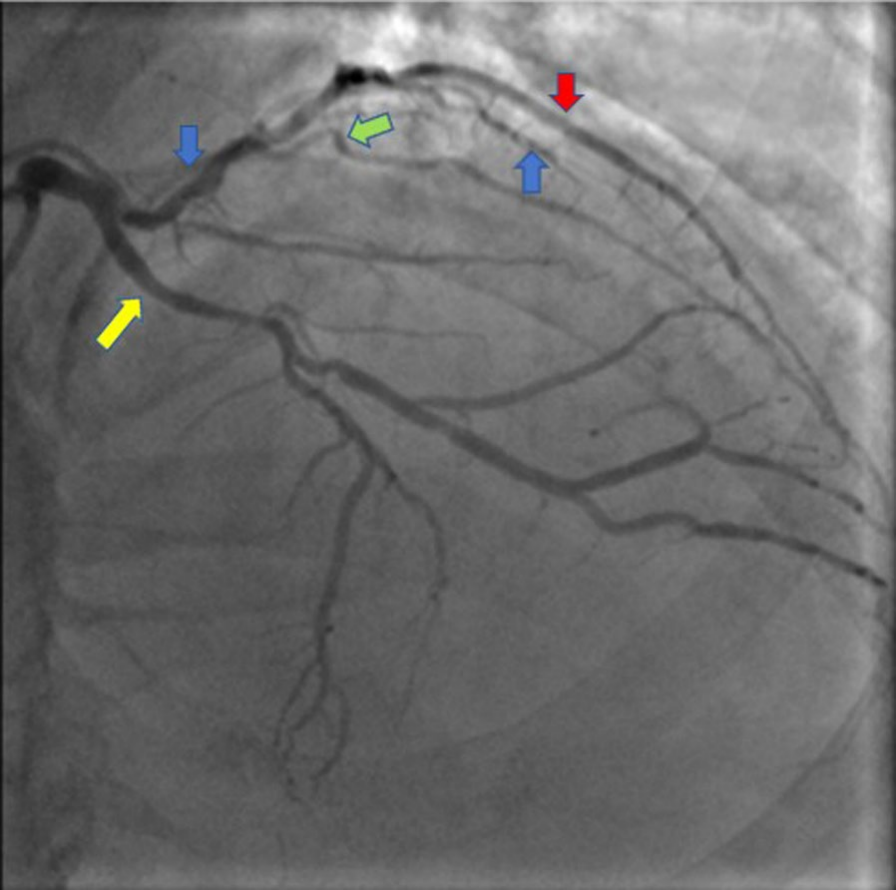
Coronary angiography RAO Caudal view. The left main coronary artery bifurcates into short left anterior descending artery (blue arrow) and left circumflex coronary artery (yellow arrow). The short LAD gives off the first septal branch (green arrow). Short LAD terminates after giving off diagonal branch *red arrow). A large diagonal branch (red arrow) arising from the LAD continues on towards the apex

**Fig. 3:**
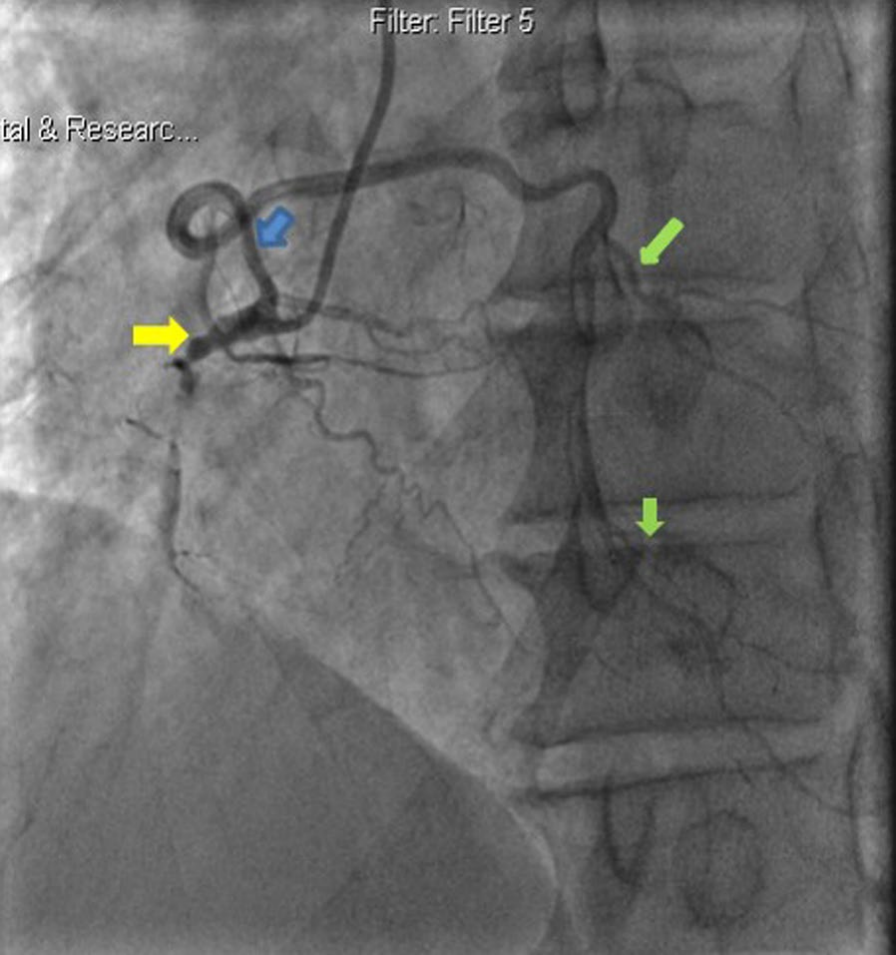
Coronary angiography in LAO view. RCA is sub-totally occluded from its proximal part with TIMI I flow (yellow arrow). The long LAD (blue arrow) arises from the proximal RCA and gives off diagonal branches (green arrow)

**Fig. 4:**
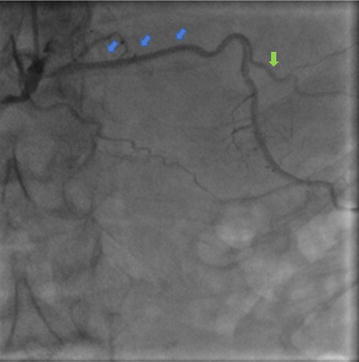
Coronary angiography showing long course of proximal part of long LAD (blue arrows) as it traverses across towards the AIVS. A diagonal branch arising from long LAD is also seen (green arrow)

Coronary angiography revealed a dual LAD of anomalous origin. The short LAD giving off the first septal branch arose from the left main coronary artery (LMCA) (Figs. 1, 2), and terminated after giving off a large diagonal branch, which continued further down towards the apex (Fig. 2). The left circumflex artery (LCx) had a normal origin from the LMCA and followed a normal course (Figs. 1, 2). There was mild to moderate disease in the LCx just before the first obtuse marginal branch. The long LAD arose from the proximal RCA, and after traversing a distance, arrived at the AIVS (Figs. 3, 4). The long LAD gave off diagonal branches and terminated at the apex (Figs. 3, 4). The RCA was totally occluded from its early mid part (Fig. 2) and well-collateralized with retrograde flow from the left system (Fig. 1). This pattern of a short LAD arising from LMCA and terminating before reaching the apex, and a long LAD arising from the proximal RCA and coursing towards the apex, is consistent with a type IV dual LAD which, to the best our knowledge, has not been previously reported in a Bangladeshi subject.

We attributed his symptoms to a prior inferior MI (which possibly occurred when he developed chest pain 6 months ago) and subsequent development of ischaemic secondary mitral regurgitation (MR) owing to a totally occluded RCA. He was offered percutaneous revascularisation and further computerized tomography (CT) evaluation of the anomalous LAD, which he declined due to financial constraints. He was discharged on optimal medical therapy consisting of dual antiplatelet therapy, stains, nitrates, diuretics, beta blockers and ACE inhibitors and was symptomatically well at subsequent follow up.

## Discussion and conclusions

Congenital anomalies of the coronary arteries occur with a reported incidence of 0.3 to 1.3% of the population undergoing a coronary angiography procedure [5]. Yamanaka et al. reported that the prevalence of dual LAD is 1% in patients of all coronary anomalies [3]. Duplication of LAD is a rare anomaly and has been categorized into 4 angiographic subtypes based on the origin, course, and termination of the short and long LAD by Spindola-Franco et al. [3].

- Type I: The short LAD runs in the AIVS and is generally the source of all major proximal septal perforators. The long LAD also runs in the AIVS, descending on the left ventricular side of the AIVS, reentering the distal AIVS to reach the apex.
- Type II: The short LAD is the same as in type 1, but the long LAD descends over the right ventricular side before reentering the AIVS.
- Type III: The short LAD is consistent with that in types 1 and 2. The long LAD travels intramyocardially in the ventricular septum.
- Type IV: The short LAD originates from the LMCA. The major septal perforators and the diagonal branches originate from this vessel. The long LAD arises from the RCA. It is extremely rare among the four types.

Some authors have further subdivided this type according to the course of the long LAD as pre-pulmonic, intra-myocardial and inter-arterial [4]. Our case is a variant of type IV LAD, and is the first such coronary anomaly described in a Bangladeshi subject. In our case, there was an apparent explanation for the patients’ symptoms: the occluded RCA from a prior MI. However, the susceptibility of the dual LAD anomaly to coronary artery disease (CAD) is of clinical interest warranting prospective studies.

It is important to be aware of the variations of dual LAD in order to avoid misinterpretation of coronary angiography and subsequent complications related to coronary interventions [2, 4, 6]. Coronary angiography provides valuable information about the degree of stenosis and involvement of other coronary arteries. Nevertheless, CT coronary angiography is advocated for further evaluation of anomalous coronaries, particularly in order to discern the exact course of the long LAD, arising from the RCA [7, 8]. Unfortunately, CT coronary angiography was not performed in our case, due to financial constraints of the patient. Furthermore, as the patient manifested at an elderly age with a clear explanation for his symptoms, we concluded that the anomalous dual LAD is of a benign nature, not responsible for his symptoms. The duplication of LAD is indeed, an incidental finding, unlikely to be a contributing factor of his symptoms, given the obvious offending event of an inferior myocardial infarction as evidenced by a totally occluded RCA. Furthermore, if the LAD indeed were to be the cause of his symptoms, there should have been an earlier onset of symptoms occurring at a younger age.

Some authors prefer to categorize coronary artery anomalies (CAA) only as “major,” “severe,” “important,” or “hemodynamically significant” anomalies versus “minor” ones [1]. The CAA described in this case is very likely a minor one, as the patient remained asymptomatic until his old age, where he presented with MI with an occluded RCA identified as the culprit lesion.

We describe a case with unique variation of dual LAD type IV, which shows a separate origin of a short LAD arising from LMCA up to first septal and diagonal branch, and long LAD originating from RCA. Coronary Angiography plays an essential role to determine this coronary anomaly, and a vast number of them are detected incidentally on routine angiography for chest pain due to involvement of other coronary arteries. Fortunately, most of them run a benign course. This was indeed the feature of our elderly patient, who became symptomatic only following total occlusion of an atherosclerotic RCA, which originated from normal right anterior sinus of Valsalva with subsequent development of ischemic MR. However, the long-term prognosis of dual LAD remains unknown, and prospective studies on the susceptibility of these coronary anomalies to CAD are warranted.

## Abbreviations

AIVS: anterior interventricular septum
CAA: coronary artery anomaly
CAD: coronary artery disease
CT: computerized tomography
LAD: left anterior descending
LCX: left circumflex
LMCA: left main coronary artery
RCA: right coronary artery
